# The Role of the Mycobiome in Women’s Health

**DOI:** 10.3390/jof9030348

**Published:** 2023-03-12

**Authors:** Michelle Marie Esposito, Savannah Patsakos, Larisa Borruso

**Affiliations:** 1 Department of Biology, College of Staten Island, City University of New York, 2800 Victory Blvd, Staten Island, NY 10314, USA; 2 PhD Program in Biology, The Graduate Center, City University of New York, New York, NY 10016, USA; 3 Macaulay Honors College, City University of New York, New York, NY 10023, USA

**Keywords:** mycobiome, breastmilk, candida, dysbiosis, synergistic

## Abstract

Although the human bacteriome and virome have gained a great deal of attention over the years, the human mycobiome has been far more neglected despite having significant value and implications in human health. In women, mycobiome profiles in breastmilk, vaginal regions, the gut, skin, and the oral cavity can provide insight into women’s health, diseases, and microbiome dysbiosis. Analyses of mycobiome composition under factors, such as health, age, diet, weight, and drug exposure (including antibiotic therapies), help to elucidate the various roles of women’s mycobiome in homeostasis, microbiome interactions (synergistic and antagonistic), and health. This review summarizes the most recent updates to mycobiome knowledge in these critical areas.

## 1. Introduction

The human mycobiome focuses not only on the collection of fungi distributed throughout the human body but also the interactions between those fungi and other microbes, as well as the host itself [1]. The overall microbiome, which includes the mycobiome, has been found to be critical to various aspects of human biology, including metabolism, pathogenesis, energy pathways, immunity, neurology, and health [1]. The human mycobiome, like the microbiome, is not a static system but instead highly variable throughout life, both at the intra- and inter-individual levels [2,3]. Colonization begins directly from birth and continues to grow and fluctuate with factors such as exercise, daily activities, nutrition, age, sex, disease, and medical therapies (especially the use of antibiotics and antifungals) [2,4]. In this review, we will specifically focus on the role of the mycobiome in women’s health and explore the various factors and implications involved in this lesser-studied part of the human microbiome. We will focus on the oral, breastmilk, skin, gut, and vaginal mycobiomes, as well as the consequences of dysbiosis in these areas.

## 2. Oral Mycobiome

Like many routes of entry of the body, the oral cavity is heavily protected by various enzymatic components of the immune system, including immunoglobulins, chaperokine heat shock proteins, lysozyme, amylase, mucins, and peroxidases, just to name a few [5]. Despite these heavily protective mechanisms, many bacteria, viruses, and fungi survive this region as part of the normal flora or microbiome of the human body [6]. Although there is a lack of research on the oral mycobiome specific to women, it is still important to explore this region of the mycobiome when examining any individual’s fungal composition. A study found a total of 85 fungal genera in the oral mycobiome, 74 of which were culturable and 11 non-cultural [7]. The most frequent genera included *Candida*, *Cladosporium*, *Aureobasidium*, *Saccharomycetales*, *Aspergillus*, *Fusarium*, and *Cryptococcus*, of which four of these predominant genera are known human pathogens [7]. While *Candida* and *Pichia* tend to be the most commonly explored dominant fungi of the oral region, other genera have emerged as demonstrating surprisingly high prevalence and abundance [8]. Of interest was the discovery of a high presence of the genus *Malassezia* [8]. *Malassezia* was previously known for its role as a pathogen on the skin, then reported as the predominant fungi of nostrils and the back of the head and ear region, prior to being identified as a strong commensal colonizer of the oral cavity [8].

It has been shown that various factors can influence the composition of these normal flora organisms, such as pharmaceutical therapies or disease states in the host [6]. In one study comparing uninfected versus HIV-infected individuals, for instance, it was shown that despite the core oral mycobiome being dominated by *Candida* species in both groups, an overall shift in abundance occurred in the HIV-infected individuals [9]. Candida albicans represented 58% of the uninfected oral mycobiome but 83% of the HIV-infected oral mycobiome, with a decrease represented in the abundance of *Pichia*, which was deemed a consequence of antagonistic effects [9]. Lifestyle choices are another factor that can affect the oral mycobiome, such as smokeless tobacco use, which has been shown to significantly decrease the richness and diversity of the mycobiome, causing a dominance of the genus *Pichia*, similar to the HIV study results, and leading to dysbiosis linked to oral cancer [10]. Even factors such as saliva levels, oral pH, and, interestingly, denture use have all been linked to oral mycobiome composition shifts, with low salivary flow, low pH, and the presence of dentures all being associated with an increase in *Candida* species [11,12]. BMI in elderly subjects has also been demonstrated as a factor influencing composition, as *Candida glabrata* and *Candida dubliniensis* were associated with elderly patients of lower BMI, which may be associated with an immunocompromised status and susceptibility to candidiasis [12]. One factor studied, specific to females, has been postpartum analyses of the oral mycobiome [13]. Not only was an increase in diversity observed in the postpartum group but also in certain genera, such as *Stachybotrys*, *Geotrichum*, *Talaromyces*, *Leucosporidium*, *Acremonium*, *Wallemia*, *Eupenicillium*, *Septoria*, *Zymoseptoria*, *Coniosporium*, *Phialophora*, and *Mycosphaerella*, which were only detected in the postpartum group [13]. Gingivitis or dental caries further modified the composition of the genera in the postpartum group, as did preterm low-weight birth [13]. Women who had preterm low-weight birth demonstrated increases in *Saccharomyces*, *Candida*, *Hyphodontia*, and *Malassezia* with a decrease in richness, but it should be noted that there was a small sample size used in this study, so it would be of great value if further studies were carried out with more pre- and postpartum women to confirm these findings [13].

While the mycobiome tends to be less studied than its bacterial counterpart, some studies have now compared changes in the two microbiomes to better understand the various factors influencing their patterns [14]. Such comparisons may not only help in the understanding of these critical communities but also may elucidate interactions that exist between them [14]. In one large study of 664 healthy Chinese adults, it was demonstrated that sex and age have a significant impact on the oral microbiome composition, while sex and age have a lesser impact on the oral mycobiome [14]. Instead, the oral mycobiome was more impacted by education level, diet factors such as fruit and vegetable consumption, and bleeding gums, which are listed in decreasing order of influence [14]. In subjects with a higher level of education or increased fruit and vegetable consumption, a decrease in *Candida parapsilosis* colonization was observed [14]. *Candida parapsilosis* is a problematic opportunistic pathogen known as a leading cause of invasive mycoses, especially in neonates and patients in intensive care units [15,16]. While education level may seem like an odd factor influencing microbial composition at first glance, it is hypothesized that one reason for this connection may be a difference in diet choices when an individual has a more extensive educational background [14]. Interestingly, the bacterial oral microbiome has also been studied in relation to hormonal cycles and menopausal women [17], but no study has explored these factors for the oral mycobiome. Such a study comparing hormonal influence on oral bacterial and fungal microbiomes would have potential value, as a study on the pathogenic oral fungi, *Paracoccidioides brasiliensis*, already showed that a significant positive correlation exists between estrogen receptors and the number of fungi present in women with oral paracoccidioidomycosis [18].

## 3. Breastmilk Mycobiome

One of the most well-known yet complex features of the female mycobiome is the one present in breastmilk. Breastmilk serves as a direct link between the mother and the infant after birth, guiding the baby’s immune system during development. Breastmilk especially impacts an infant’s gut mycobiome as it is the earliest source of food and nutrients [19]. It is vital to recognize that fungi within the human gut can regulate bodily inflammation, immunity, and overall metabolism, causing breastmilk to play a large role in one’s future health [19]. The University of Minnesota collected samples of breastmilk from mothers who delivered preterm and isolated the DNA [19]. The fungal primers (UN1 and UN2) were used to amplify the fungal DNA, and cell-free fungal DNA was also evaluated, then sequenced at the university [19]. All of the breastmilk samples that were used contained fungal sequences, which were present in fungal cells rather than cell-free DNA [19]. The taxa of highest abundance were *Candida albicans*, *Candida parapsilosis*, *Cryptococcus neoformans*, *Saccharomyces cerevisiae*, and *Candida glabrata* [19]. Both *Candida glabrata* and *Cryptococcus neoformans* are the taxa of fungi that are correlated with human disease, showing great relevance to newborn health and mycobiome development [19]. It is also important to recognize that food intake can greatly influence the mycobiome of breastmilk, which is directly received by the infant. Mycotoxins are secondary metabolites produced by fungi present in some foods such as crops, which are toxic to human health [20]. Researchers collected mature breastmilk samples from a variety of countries, including Spain, Germany, and Bangladesh, isolating the fungi to identify their presence and abundance [20]. Not only were mycotoxins present but other toxins, such as beauvericin, enniatins, and aurofusarin, were also found present in the samples [20]. The abundance and diversity of fungi present in breastmilk led to experiments concerning the effectivity and usefulness of using breastmilk to combat pathogens. A study conducted by Mohamed T. El-Saadony and colleagues isolated lactic acid bacteria from samples of female breastmilk in order to create selenium nanoparticles (SeNPs) [21]. These were then used to treat different fungi that often cause human infection, including *Candida* and *Fusarium* [21]. The antifungal activity was portrayed via plating, where *Candida* and *Fusarium* were plated on agar, discs treated with the SeNPs were placed on the fungi, and their zones of inhibition were measured [21]. The particles developed from the lactic acid bacteria in breastmilk successfully inhibited the growth of both of these fungal species, portraying sufficient zones of inhibition on the agar plates, thereby portraying a new treatment against animal fungi pathogens [21]. This experiment portrays that breastmilk not only has an abundance of important fungi, but it also has vital bacteria that can be used to treat actual fungal infections. The diverse nature of breastmilk makes it an extremely beneficial scientific tool, especially in medicinal treatment.

## 4. Skin Mycobiome

While the microbiome of the skin also includes bacteria, microeukaryotes, and viruses, it is fungi that represents the second most prevalent group of the skin microbiome [22]. The fungal skin mycobiome tends to be dominated by *Malassezia*, *Candida*, *Cladosporium*, *Fusarium*, and *Cryptococcus* [22]. As with the oral mycobiome, various factors can influence the compositional changes that occur in the community residing on the skin. For instance, age and sex appear to play a role in the colonization of the skin, with microbiome transitions observed during the sexual maturation stages of puberty [22]. It has been suggested that the connection between mycobiome community changes during puberty are controlled by sex hormone secretions that increase sebaceous gland activities [22]. Between the sexes, female children have been found to have a greater dominance of *Malassezia* in their skin mycobiome [22]. Furthermore, age plays a major role in these microbial communities, with a significant decline in fungal diversity associated with age beyond puberty and above 60 years [22].

Fungal diversity has also been shown to vary in cases of sensitive skin syndrome versus non-sensitive [23]. One study of the skin mycobiome of Korean women subjects demonstrated greater phylogenetic diversity, with increases in *Lactobacillus* and *Mucor racemosus* and a decrease in *Malassezia restricta* in sensitive skin subjects [23]. The increase in fungal diversity in sensitive skin sufferers is quite distinctive as a usually greater diversity of bacterial microbiomes has been associated with healthy individuals [23]. The analyses of these women also demonstrated interactions exist between the fungal skin mycobiome and the bacterial skin microbiome, with negative interactions between *Lactobacillus* and *M. restricta* in healthy individuals and *Delftia* and *Bacteroides caccae* in sensitive skin individuals who suffered a decrease in *M. restricta* [23]. Understanding these interactions and the varying compositions in the microbiomes of sensitive skin sufferers can help to draw attention to certain products of the microbes that may be responsible for exacerbating sensitivity or other health issues. For instance, *Lactobacillus*, mentioned in those interactions, is known for modifying the pH of their surroundings, which could, in turn, affect the environment when their concentrations fluctuate, such as the dysbiosis and risk of urogenital or reproductive health issues when lactobacilli concentrations drop in vaginal environments [23,24].

Additionally, in the case of women’s health, it has been found that the mode of birthing by the mother can impact the skin mycobiome composition or diversity [25]. Through sequencing analyses, it has been found that vaginally born children exhibit skin mycobiomes with vagina-associated fungi, such as *Candida* or *Rhodotorula*, whereas cesarean-delivered children include mycobiomes with a greater presence of skin or airborne-associated genera, such as *Malassezia* and *Alternaria* [25]. While that may seem rather obvious, other findings demonstrate more surprising findings, such as cesarean mycobiomes exhibiting more niche-based processes, fragile networks, and unchanged dissimilarity not observed in vaginally born mycobiomes [25]. These findings demonstrate that it is of great importance not only for the adult host to study mycobiomes but that through analyses of mothers, information can be gained about the influences of the early development of mycobiomes, as well as hygiene-based analyses of the microbiome [25]. Furthermore, neonatal fungal information helps prevent or reduce early exposure or incidence of infections in newborns [26].

## 5. Gut Mycobiome

The gut mycobiome is one of the most vital and diverse microbial systems within the human body [27]. Due to its diversity, the gut mycobiome plays a variety of functional roles, including those of homeostasis and immunity [28]. A vital aspect of the gastrointestinal tract is the presence of fungi, which, especially in dysbiosis, influence the onset of different diseases and disorders, including cancers and autoimmunity [29]. In healthy individuals, the common fungi in the gastrointestinal tract include *Candida*, *Saccharomyces*, *Malassezia*, and *Cladosporium*, where *Candida* spp. are often the most abundant [30,31]. These fungi develop important interdependent relationships with the bacteria present in the gut, developing mutualism to provide the best environment for host survival [32]. Factors such as diet, genetics, and lifestyle greatly impact one’s mycobiome and its development over time [33]. Disrupting the fungal environment has been shown to cause irritable bowel syndrome, coeliac disease, and pancreatic disorders, displaying the importance that these fungi play in maintaining one’s health [34]. Disruptions by viruses such as hepatitis B and HIV even affect systems outside of the gastrointestinal tract, specifically the immune and hepatic systems, which can cause extensive disease and morbidity [35,36]. There are harmful fungi that can disrupt the gastrointestinal tract that have also been linked to disease, such as how an increase in *Malassezia restricta* is linked with Crohn’s disease and inflammatory bowel disease [37,38]. Despite alterations such as these, most fungi of the mycobiome are beneficial and extremely useful for the host [39]. For example, the intestinal fungi, *C. albicans* and *S. cerevisiae*, can prevent one’s susceptibility to further infections, such as colitis, after taking antibiotics that caused a reduction in gut bacteria [39].

As previously discussed, the gut mycobiome has a relationship with other bodily systems, such as the immune system [39]. A key element for this relationship is through the lymphoid tissue that interacts with the gut itself [40]. On top of diet playing an influential role in the status and composition of the gut, psychological stress plays a crucial role as well, especially for pregnant women [40]. Psychological stress can also alter the gut mycobiome of the developing fetus, which can be harmful, causing behavioral disorders and neuroimmune issues [40]. Since the mother’s body is directly responsible for the growth of the fetus, medicinal treatments such as antibiotics can also alter the fetus’ gut [40]. A reduction in microbes so early in development can directly inhibit the development of both innate and adaptive immunity for the infant, both of which are impacted by the gut mycobiome [40]. It is evident that the more stress the pregnant female is under, the less diverse the microbiome becomes [40]. This lowered diversity is correlated with a decreased immune response for both the mother and fetus, portraying the vitality of gut mycobiome diversity [40]. Not only does this portray an intricate relationship between the gut and major bodily systems, such as the immune system, but it ties the status of one’s gut to one’s stress/mental status as well [40]. For example, there is even evidence that an unhealthy gut mycobiome has been related to irritable bowel syndrome (IBS), which is often comorbid with psychiatric illnesses, anxiety, and depression [41]. IBS often results in gastrointestinal inflammation and mycobacterium dysbiosis [41]. Psychiatric disorders can result from this dysbiosis due to the promotion of pro-inflammatory communication with bodily systems, specifically with the brain’s stress system, such as the hypothalamic–pituitary–adrenal (HPA) axis [41]. This unusual pro-inflammatory communication has been directly tied to increased cortisol production by the brain, and these high levels are often found in individuals suffering from anxiety and depression [41]. Some of the bacteria in the gastrointestinal tract even produce neurotransmitters and neuropeptides that play a direct role in mental health, including that of serotonin [41]. The gut bacterial composition was also measured for patients with IBS, major depressive disorder, and anxiety disorders, showing that all three portrayed lower alpha diversity, higher levels of Proteobacteria, and higher levels of *Escherichia*/*Shigella* [41]. *Escherichia* and *Shigella* often produce exotoxins, which are known to cause an increase in inflammation, which is detected by the brain through communication, such as via the gut–brain axis [41]. Even though the cause and effect of these comorbidities are unclear, it is important to recognize the relationships that gut microbiota have with psychological conditions in order to exemplify their importance in the human body [41].

## 6. Vaginal Mycobiome

Before recently, most studies of the vaginal mycobiome revolved around *Candida albicans*, which is known to be a leading cause of vaginal infection [42]. Although *Candida albicans* is estimated to make up over 70% of fungi found in the vaginal mycobiome, a number of non-albicans species are also present in smaller numbers [42]. These non-albicans species include *C. krusei*, *C. parapsilosis*, *C. tropiclis*, *C. glabrata*, *C. guilliermondii*, *C. pseudotropicalis*, *C. stellatoidea*, and others [42]. The most common fungi other than *Candida* in the vagina mycobiome are *Saccharomycetales*, *Davidiellaceae*, *Cadosporium*, and *Pichia* [43,44]. Existing studies do not support strain tropism for *Candida*-induced infections on the grounds that strains isolated from patients who show signs of vulvovaginal candidiasis (VVC) appear identical to strains collected from individuals who show no symptoms of VVC [45]. It is estimated that roughly 10–20% of healthy women harbor commensal *Candida* fungal colonies that cause no physical symptoms in the vaginal area [46].

A 2012 study identified 3 phyla of fungi from 28 successfully identified OTUs present in the vaginal area [47]. The study relied on 18S rRNA gene clone sequence libraries and examined the mycobiota of healthy women, women with allergic rhinitis (AR), women with recurrent vaginal candidiasis (RVC), and women with RVC complicated by AR [47]. The phyla included Ascomycota, which made up 78.6% of the identified OTUs; Basidiomycetes, which made up 17.8% of the identified OTUs; and Oomycetes, which made up 3.6% of the identified OTUs [47]. *Candida* was the primary genera of present Ascomycota [47]. This same study concluded that women with RVC and AR have higher populations of *C. albicans* in the vaginal area as compared to healthy women and that women with RVC have lower populations of *S. cerevisiae* in the vaginal area as compared to healthy women [47]. Additionally, the study found a general increase in the diversity of vaginal fungal flora in women with RVC and AR [47]. This led the researchers to the conclusion that allergic reactions in the vagina could alter the fungal flora of patients affected by RVC and AR [47].

In the first next-generation study of vaginal mycobiota, researchers in Estonia calculated the relative abundance of *Candida* species in the vaginal area at 36.9% and rates of vaginal colonization with *Candida* at 64.5% [43]. The Estonian study relied on the amplification of fungal internal transcribed spacer-1 (ITS-1) regions using 454 Life Sciences pyrosequencing. The researchers noted that the prevalence of *Candida* ascertained in their study was significantly higher than the prevalence rates of earlier studies [43]. The study identified two phyla of fungi present, including *Ascomycota*, which made up 58% of identified sequences, and *Basidiomycota*, which made up only 3%. A total of 82% of the *Ascomycota* OTUs identified as *Candida* belonged to *C. albicans* [43]. An obstacle in adequately describing the totality of the data was the large number of unspecified OTUs, in which no taxonomic assignment lower than kingdom was available. This made up a large portion of the data at 38% [43]. This study, therefore, highlights an important problem in the study of mycobiota, which is the low number of fungal species represented in reference databases. Compared to the database used for bacterial 16S rDNA, fungal databases are relatively undeveloped and sometimes unsuitable for the study of fungi present in humans [48].

It should be noted that non-*albicans Candida* OTUs have also been identified as vaginal pathogens. *C. parapsilosis* and *C*. *krusei* have both been known to cause vaginitis, with the latter predominantly affecting older women [49,50]. Still, these fungi, like *C. albicans*, are commonly found in the vaginal mycobiota of healthy women [43].

## 7. Mycobiome Dysbiosis

Although it has been touched upon in the previous sections of this review, it is important to reiterate the significance of the mycobiome and how dysbiosis in any part of a microbiome can have severe consequences on host health, as well as other communities within the overall microbiome [51]. While the contributions of bacterial microbiome dysbiosis toward disease have been thoroughly studied, mycobiome dysbiosis has been heavily neglected [52]. Dysbiosis of the mycobiome occurs from various factors, but some common ones include diet, skin contamination, lifestyle activities, medical treatments or pharmaceuticals, environmental factors, and hygiene practices [53].

One area of mycobiome dysbiosis that has important implications for women is in the vaginal microbiota community [52]. In a study of intrauterine adhesion (IUA) disease, it was found that certain fungal genera, such as *Filobasidium* and *Exophiala*, are more enriched in IUA samples versus healthy subjects [52]. It has also been shown that fungal–bacterial patterns and interactions exist in this region. *Ascomycota* and *Basidiomycota*, for instance, demonstrate correlations with *Proteobacteria* in cervical canal IUA samples, while a negative association was observed between *Prevotella bivia* and *Candida maltose* [52]. In the healthy subjects, but not the IUA subjects, a negative correlation between *C. parapsilosis* and *Cutaneotrichosporon jirovecii* was also observed [52]. While most people assume fungi have a pathogenic impact on bodies, interestingly, it has also been observed that the presence of particular strains, such as *C. parapsilosis*, actually has a protective impact on certain disease progressions, such as IUA [52]. In a rat model of IUA, a reduction in inflammation and fibrosis was observed in the presence of *C. parapsilosis* [52]. This protective ability was also observed in gut studies, where *C. parapsilosis* was shown to protect against damage that would be caused by *Candida albicans* in intestinal epithelial cells [54]. When coadministered, *C*. *parapsilosis* reduced infection rates and mortality in animal models [54]. A better understanding of the role of fungi in the pathogenesis or protection of IUA cases could have a significant impact on the health and wellbeing of women, as IUA pathogenesis is linked to pregnancy terminations, hypomenorrhea, amenorrhea, and infertility with partial or total obstruction of the uterine cavity and/or cervical canal [52,55]. Beyond IUA, changes in the vaginal mycobiome composition can result in the destruction of important bacterial normal flora, such as Lactobacilli, which, in turn, triggers a cascade of complications as Lactobacilli are critical for their antifungal properties and antagonistic competition [44]. Changes of this nature can lead to diseased states, such as candidiasis [44]. Dysbiosis and synergistic bacterial interactions with native vaginal *Candida*, specifically with Streptococcus group B and E. coli, can also be a problem as it has been linked to preterm birth, low birth weight, and sepsis [44,56]. Due to the interactions that exist between bacteria and fungi, Lactobacilli-containing probiotics have been considered a potential treatment and preventative supplement for fungal vaginal dysbiosis [57]. These probiotics have proven useful for bacterial vaginosis but not for vulvovaginal candidiasis and require more research to be performed before they become mainstream in the pharmaceutical industry [57].

Changes in the mycobiome have also been implicated in the pathogenesis and clinical presentations of skin diseases, such as atopic dermatitis [58]. The primary fungal imbalances observed in head and neck variants of atopic dermatitis have been in the rates of *Malassezia* and *Candida*, and appear to be associated with fungal antigens producing robust immune responses, sensitization, and skin lesions [58]. Antifungal immune system responses include C-type lectin receptors, IL-1β, and inflammasomes [59]. In atopic dermatitis skin lesions, it has been found that *Malassezia* levels are decreased, and filamentous fungi are increased, along with a positive correlation between *Candida* and Staphylococcus [60]. Understanding and elucidating mycobiome implications of this nature could help in the development of more effective treatments for the ailments [58].

Overall, fungal mycobiome dysbiosis can have severe consequences for the host, with studies finding fungal composition differences in head and neck cancer carcinoma, colorectal carcinoma, and pancreatic ductal adenocarcinoma, particularly with an increased presence of *Malassezia* correlated with onset and progression of the diseased state of colorectal and pancreatic cancers [61]. On the other hand, some members of the mycobiome can be beneficial, with oral fungi, such as *Schizophyllum*, linked to anticancer potential [61]. As early as 1969, the glucan schizophyllan produced by *Schizophyllum* showed anti-tumor abilities on subcutaneously implanted tumors of sarcoma-37, sarcoma-180, Ehrlich carcinoma, and Yoshida sarcoma [62]. More recently, *Schizophyllum commune*, as well as *Geopora sumneriana*, edible mushrooms have been used to synthesize anticancer and antimicrobial silver nanoparticles that demonstrate potential against breast, lung, colon, and liver cell lines, as well as against *Pseudomonas aeruginosa*, *Klebsiella pneumonia*, *Staphylococcus aureus*, *Enterococcus faecalis*, and fungal *Candida albicans* and *Candida utilis* [63]. Glucan expressed from *Schizophyllum* presents as a valuable potential treatment in cancers and disease [64]. MTT assays have shown the *Schizophyllum* glucan is not cytotoxic, and yet is capable of increasing macrophage activation and immune responses while also leading to observed noticeable decreases in breast cancer cell proliferation and tumor volume through the induction of apoptosis [64].

Gut and intestinal mycobiome dysbiosis is also an area of concern [65]. It might be assumed that gut microbes are just involved in digestion, but healthy or proper gut mycobiome communities appear to also be significant for proper immune function as well [65]. In one study using a mouse model, it was determined that prolonged antifungal oral drug applications resulted in an increase in the severity of colitis and an increase in the development of allergic airway disease [65]. When it was observed that *Candida* was reduced with the antifungal, but three strains of fungi increased (*Aspergillus*, *Wallemia*, and *Epicoccum*), supplementation with the three identified strains then resulted in the same allergic disease observed during the antifungal treatments [65]. This demonstrated to researchers that dysbiosis of critical fungal commensal communities in the gut was able to initiate harmful immune responses and increase disease states [65]. Significant gut fungal dysbiosis is also associated with cirrhosis in conjunction with bacterial dysbiosis, allowing for *Bacteroidetes*/*Ascomycota* ratios to be used to predict 90-day hospitalizations [66]. Ascomycota changes in gut mycobiomes have also been associated with Crohn’s disease flares [67]. While overall fungal volumes increased in Crohn’s patients, noticeable dominance of colonic fungal microbiota by Basidiomycota and Ascomycota was observed [67]. Other noticeable alterations in the fungal microbiome of Crohn’s patients is the association of *Saccharomyces cerevisiae* and *Filobasidium uniguttulatum* with non-inflamed mucosa versus the association of *Xylariales* with inflamed mucosa [67]. Many times, emphasis is placed on bacterial alterations in the microbiota during pathogenesis and, thus, in treatment, but rarely has the fungal microbiota been explored in this manner [67]. Overall, mycobiome dysbiosis can have a significant impact on the overall health and wellbeing of the human host (Table 1).

## 8. Conclusions

As can be seen by a major theme in this review, fungal microbiota has been greatly overlooked throughout the years, as bacterial microbiome communities have frequently garnered most of the attention [68]. As demonstrated, however, the mycobiome has significant connections to the bacterial microbiome and plays major roles in pathogenesis, immunity, and overall health, even holding some anticancer properties [69,70]. For instance, fungal mycobiome colonizers, such as *C. albicans*, serve as critical regulators of immune system development and function during eubiosis in immune priming [71]. A signal cascade is triggered in which dendritic cells sense fungi and initiate helper T-cell responses, antifungal IgG, and cytokines that support the production of immune key players, such as mucus, defensins, and immunoregulatory cytokines [71]. Fungi also release metabolites, such as candidalysin, which serve as a cytotoxin that promotes antifungal inflammatory responses and immune system cascades [71]. Additionally, it has been observed that changes in the mycobiome, which then change the bacterial microbiome, can influence critical metabolites within the body, such as short-chain fatty acids, organic acids, taurine, butyrate, and succinate [72]. Furthermore, metabolites produced directly by mycobiome fungal colonizers have been linked to shaping host health with metabolic diseases and cancers associated with changes in fungal metabolites [53]. One metabolite highlighted in studies of the mycobiome is N-acetyl-L-glutamic acid, which has hypotensive consequences [53]. The fungi of the mycobiome are also involved in metabolizing biomolecules, such as lipids, carbohydrates, and proteins, while also producing a series of secondary metabolites, such as acids, toxins, and sugars [53]. These fungal metabolites have been separated into classes that include polyketide synthases (PKSs), non-ribosomal peptide synthases (NRPSs), fatty acid derivatives, terpenoids, and steroids [53]. Understanding the metabolites of the fungi in the mycobiome has been associated with studying various health issues, including Alzheimer’s disease and cancer [53].

**Figure 1:**
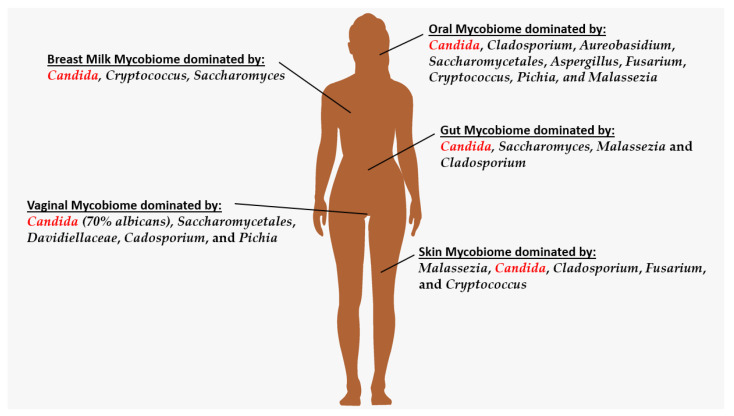
Summary of the dominant fungi present in the oral, breastmilk, skin, gut, and vaginal mycobiomes, with *Candida*, specifically *Candida albicans*, the most abundant (in red font) of the fungi native to the human body.

Although there is great diversity in the fungi associated with the human body, including different dominant genera in each section explored in this review, such as the robustly diverse oral mycobiome dominated by *Candida*, *Cladosporium*, *Aureobasidium*, *Saccharomycetales*, *Aspergillus*, *Fusarium*, *Cryptococcus*, *Pichia*, and *Malassezia*, compared to less diverse breastmilk dominated by *Candida*, *Cryptococcus*, and *Saccharomyces*, one genus, *Candida*, appears dominant across the overall human body (Figure 1) [42,43,44]. In the case of the vaginal region, in fact, *Candida* has been attributed to account for over 70% of the vaginal mycobiome [42]. Understanding the fungal communities of the microbiome not only aids in the development of treatments or the characterization of pathogeneses or immunity but also provides insight into reproductive health and neonatal health [73,74]. Furthermore, studies into these microbial communities have allowed us to see that fungi of the mycobiome are not simply commensal organisms in relation to the human host, but also live in intricate symbiosis with the massive and diverse bacterial communities of the microbiome [75,76]. By having both microbial communities as part of the overall human microbiome, extensive control and protection in our daily lives occur without us even being aware, such as how bacteria keep commensal *Candida* from pathogenesis via prevention of yeast–hyphal transition and increased epithelial integrity [77]. While often understudied, the human mycobiome clearly has importance in health and disease and, thus, deserves a greater spotlight than currently is afforded to it [78].

## Figures and Tables

**Table 1:** Summary of the significance of mycobiome dysbiosis on the human host.

Region of Dysbiosis	Significance
**Vaginal**	*Filobasidium* and *Exophiala* are more enriched in IUA samples Reduction in inflammation and fibrosis in IUA samples in the presence of *C. parapsilosis* Destruction of vaginal bacterial microbiome
**Gut**	*C. parapsilosis* protects against damage of *Candida* to intestinal epithelial cells Antifungal oral drugs increase severity of colitis and development of allergic airway disease Associated with cirrhosis, Crohn’s disease, and bacterial dysbiosis
**Skin**	Implicated in pathogenesis and clinical presentation of disease states, such as atopic dermatitis Production of robust immune responses, sensitization, and skin lesions
**Overall** **Mycobiome**	Associations with head and neck cancer, colorectal carcinoma, and pancreatic cancer Increases in some genera, such as *Schizophyllum*, have opposite effect and benefit host with anticancer and antimicrobial properties

## Data Availability

Not applicable.
